# Relationship of subclinical lung injury to chronic airway inflammation in spinocerebellar ataxia type 3

**DOI:** 10.1186/s13023-026-04306-5

**Published:** 2026-03-09

**Authors:** Xiao-Ting Lv, Wei Lin, Bei-Ning Ye, Ze-Wei Zhang, Han Lin, Jia-Yi Zhang, Zi-Ying Zhou, Mao-Lin Cui, Zhuo-Ying Huang, Ning Wang, Shi-Rui Gan

**Affiliations:** 1 Department of Respiratory and Critical Care Medicine, The First Affiliated Hospital of Fujian Medical University, Fuzhou, China; 2 Department of Respiratory and Critical Care Medicine, National Regional Medical Center, Binhai Campus of the First Affiliated Hospital, Fujian Medical University, Fuzhou, China; 3 Institute of Respiratory Disease, Fujian Medical University, Fuzhou, China; 4 Department of Neurology, Fujian Institute of Neurology, The First Affiliated Hospital, Fujian Medical University, Fuzhou, China; 5 Fujian Key Laboratory of Molecular Neurology, Fujian Medical University, Fuzhou, China; 6 Department of Ophthalmology, Shanghai Ninth People’s Hospital, Shanghai, China; 7 School of Basic Medical Sciences, Fujian Medical University, Fuzhou, China; 8 School of Special Education and Rehabilitation, Binzhou Medical University, Yantai, China; 9 Department of Neurology, National Regional Medical Center, Binhai Campus of the First Affiliated Hospital, Fujian Medical University, Fuzhou, 350005 China

**Keywords:** SCA3, Pulmonary function, SARA, Chronic inflammation of small airways

## Abstract

**Background:**

Spinocerebellar ataxia type 3 (SCA3) is one of the most prevalent hereditary neurodegenerative disorders, with respiratory failure being the leading cause of mortality. Nevertheless, pulmonary function in SCA3 has not been thoroughly characterized, and the underlying mechanisms remain unclear.

**Methods:**

We conducted pulmonary function tests in both patients and healthy controls, as well as in SCA3 mice and wild-type mice. In participants, we used diaphragm ultrasonography to evaluate diaphragmatic dysfunction. We examined clinical features and inflammatory biomarkers to identify independent associations with pulmonary function. We also performed histopathological and immunohistochemical analyses on lung tissues from SCA3 mice to assess the presence of chronic airway inflammation.

**Results:**

We enrolled 102 patients and 91 healthy controls for pulmonary function testing. For blood biomarker analyses, SCA3 participants with available blood data were drawn from the same patient cohort and were compared with an independent cohort of 88 age- and sex-matched healthy controls, distinct from the pulmonary-function controls. Compared with controls, patients showed significant reductions in FVC (*P* < 0.001), FEV1 (*P* < 0.001), DLco-SB (*P* = 0.015), and small-airway flow indices (*P* < 0.05), along with an increased RV/TLC ratio (*P* < 0.001), indicating subclinical pulmonary dysfunction. Impaired pulmonary function, defined according to prespecified criteria, was identified in 71/102 (69.6%) patients versus 0/91 controls (*P* < 0.001). Pulmonary impairment was associated with ataxia severity (SARA: OR = 1.196, 95% CI 1.038–1.376; *P* = 0.013). Diaphragm ultrasound showed preserved diaphragmatic function, whereas systemic inflammatory markers were associated with reduced pulmonary function, with NLR inversely correlated with MEF25 (ρ =–0.272, *P* = 0.010). In SCA3 mice (*n* = 4) compared with wild-type mice (*n* = 3), pulmonary function abnormalities and lung pathology were consistent with airway inflammatory changes.

**Conclusion:**

SCA3 is associated with early, subclinical pulmonary dysfunction that worsens with disease progression. Chronic inflammation in the small airways may be involved in this process. Our findings underscore the need for early intervention with chest physiotherapy and respiratory training as part of the clinical management of SCA3.

**Supplementary Information:**

The online version contains supplementary material available at 10.1186/s13023-026-04306-5.

## Introduction

Spinocerebellar ataxia type 3 (SCA3), also known as Machado-Joseph disease (MJD), is a progressive autosomal dominant cerebellar ataxia caused by mutations in the *ATXN3* gene. This mutation leads to the accumulation of polyglutamine (Poly Q)-expanded ataxin-3 protein within neuronal nuclei [1, 2]. Ataxin-3, encoded by *ATXN3*, functions as a deubiquitinating enzyme expressed broadly across multiple tissues and cell types [3–5]. It plays essential roles in maintaining protein homeostasis, DNA repair, cytoskeletal regulation, myogenesis, degradation of misfolded proteins, cell cycle control, and apoptosis [3, 6]. Crucially, pathological expansion occurs when the CAG repeat in *ATXN3* exceeds 56 repeats, causing an abnormal elongation of the carboxyl-terminal region of ataxin-3 and resulting in the formation of Poly Q aggregates [7–9].

Although significant advances have been made in understanding SCA3 etiology, the disease inevitably follows a progressive trajectory that results in premature death or severe disability, with a median survival of 25 years after ataxia onset [10–12]. The 10-year mortality rate among SCA3 patients ranges from 25% to 35% [13]. Research involving European cohorts has established that SCA3 affects multiple organ systems, with pulmonary complications, including impaired lung function, representing the leading cause of death in these patients [14]. Similar pulmonary dysfunction has also been observed in other neurodegenerative disorders such as amyotrophic lateral sclerosis (ALS) [15], Parkinson’s disease (PD) [16], and multiple system atrophy (MSA) [16]. In ALS, patients show a marked decrease in predicted forced vital capacity (FVC) percent, indicating compromised ventilatory capacity [15]. PD patients are prone to increased morbidity and mortality due to respiratory system abnormalities, including restrictive and obstructive ventilatory defects and dysfunction of the upper airway and intercostal muscles [17]. MSA can manifest as primary respiratory failure or dysfunction, often preceding overt motor and autonomic symptoms [18]. Despite a small study suggesting that SCA1, SCA2, and SCA3 patients may exhibit subclinical restrictive pulmonary impairment [19], no large-scale clinical evaluations of pulmonary function in SCA3 patients have been conducted. Consequently, the underlying causes of pulmonary dysfunction in SCA3 remain unclear.

The causes of impaired pulmonary function vary among neurodegenerative diseases. For instance, ALS patients may develop pulmonary dysfunction due to reduced physical activity or suboptimal care [15], whereas PD patients often experience respiratory impairment linked to upper airway and intercostal muscle abnormalities [17]. Recent evidence highlights inflammation as a critical contributor to pulmonary function decline. Circulating white blood cell (WBC) subpopulations and the neutrophil-to-lymphocyte ratio (NLR), both established inflammatory biomarkers [20, 21], have been implicated as causal factors in respiratory impairment [22, 23]. In occupationally exposed populations, elevated neutrophil, eosinophil, and basophil counts correlate with diminished lung function [22]. Studies have also shown that neutrophil-driven oxidative stress [24], facilitated by neutrophil extracellular traps (NETs), plays a central role in acute lung injury (ALI) [25, 26] and the pathogenesis of chronic obstructive pulmonary disease (COPD) [27].

In this study, we analyzed a large cohort of SCA3 patients alongside SCA3-YAC-84Q transgenic mice to evaluate pulmonary dysfunction and its relationship with clinical and genetic variables in SCA3. We further explored whether chronic airway inflammation is associated with impaired pulmonary function in this disease.

## Materials and methods

### SCA3 patients and healthy controls

We conducted a prospective, cross-sectional cohort study to examine pulmonary dysfunction and its potential clinical and genetic correlates in SCA3 patients. A total of 102 individuals with genetically confirmed SCA3 were consecutively enrolled from the Organization in South-East China for Cerebellar Ataxia Research (OSCCAR) at the Department of Neurology, First Affiliated Hospital of Fujian Medical University, between November 2021 and August 2023. All 102 SCA3 patients underwent pulmonary function testing. For blood biomarker analyses, blood data was available for 88 of these patients. These 88 individuals were drawn from the same SCA3 cohort and also completed pulmonary function testing. For comparison, we recruited 91 healthy controls matched for sex, age, and body mass index (BMI), all of whom carried normal-range CAG repeat lengths and were drawn from the same institution. These 91 healthy controls constituted the pulmonary function testing control cohort. For blood biomarker analyses, an independent cohort of 88 age- and sex-matched healthy controls was recruited and did not overlap with pulmonary function testing controls (*n* = 91). The study protocol received approval from the Ethics Committee for Medical Research at the First Affiliated Hospital of Fujian Medical University ([2019]195), and all participants provided written informed consent.

### Inclusion criteria

Participants were eligible if they met all of the following criteria: (1) genetically confirmed SCA3 with an expanded *ATXN3* CAG repeat length > 56; (2) age 18–75 years; (3) definite ataxia (SARA > 3); (4) ability to understand and complete standardized pulmonary function testing; and (5) no overt respiratory symptoms at enrollment and no clinical evidence of active pulmonary disease or severe underlying pulmonary disorders.

### Exclusion criteria

Participants were excluded if any of the following were present: (1) current pneumothorax or within one month after healing of a pneumothorax; (2) severe cardiopulmonary or vascular comorbidities, such as severe pulmonary bullae, thoracoabdominal aortic aneurysm, cardiac insufficiency, hypoxemia, or respiratory failure; (3) acute or unstable cardiac conditions, including unstable angina or recent myocardial infarction; (4) poorly controlled severe hypertension, hypertensive crisis, or severe coronary artery disease; (5) inability to cooperate due to neurological or cognitive impairment (e.g., hemiplegia, facial paralysis, recent stroke, cerebral palsy, or intellectual disability); and (6) active bleeding risk, including hemoptysis within the preceding two weeks or active gastrointestinal bleeding.

### Genotype and phenotype analyses in SCA3 patients and healthy controls

To determine CAG repeat lengths in the *ATXN3* gene, we employed polymerase chain reaction (PCR) followed by Sanger sequencing, as described previously [28]. Trained ataxia specialists and respiratory physicians conducted in-person interviews with each participant to obtain comprehensive clinical and respiratory data. Respiratory symptoms, including chronic cough, sputum production, and wheezing [29, 30], were assessed according to established clinical definitions. Subclinical pulmonary dysfunction was defined as the presence of impaired pulmonary function on PFT in participants without respiratory symptoms [31]. Pulmonary function was categorized as normal or impaired. Impairment was diagnosed when at least one prespecified abnormality was present, including obstructive ventilatory dysfunction, restrictive ventilatory disorder, mixed ventilatory disorder, small airway dysfunction, or impaired diffusion capacity, as defined below [32]. Age at onset (AAO) was defined as the age when gait disturbances were first recognized by the patient, a close relative, or a caregiver. Disease duration was calculated as the time interval between AAO and the age at which pulmonary function testing was performed. Smoking history was ascertained through a structured interview or questionnaire. For the primary analysis, it was dichotomized into “ever smoker” (including both current and former smokers) and “never smoker” (having smoked < 100 cigarettes in lifetime) [33]. We assessed ataxia severity using the Scale for the Assessment and Rating of Ataxia (SARA), which comprises eight items with a cumulative score ranging from 0 (no ataxia) to 40 (most severe ataxia) [34].

### Assessment of pulmonary function in patients and healthy controls

We performed pulmonary function tests (PFTs) on all participants using the Pulmonary Function Tester (Vyaire Medical GmbH, Bavaria, Germany). The PFT protocol included assessments of ventilatory function, pulmonary diffusion capacity, and lung volumes. We evaluated ventilatory function through both slow breathing and flow-volume loop tests, while we assessed diffusion capacity and lung volumes using the single-breath diffusion technique. All procedures were conducted with subjects seated. Each participant repeated the test maneuvers three times, with two-minute rest intervals between trials. For analysis, we used the best performance from the three attempts. Ventilatory function served as the primary component of the PFT. We measured the following parameters: FVC, forced expiratory volume in one second (FEV1), FEV1/FVC ratio, peak expiratory flow (PEF), maximal expiratory flow at 75%, 50%, and 25% of vital capacity (MEF75, MEF50, MEF25, respectively), mean mid-expiratory flow (MMEF75/25), the ratio of forced expiratory flow at 50% of vital capacity to forced inspiratory flow at the same level (FEF50%/FIF50), and maximal voluntary ventilation (MVV). To assess pulmonary diffusion function, we measured the diffusing capacity of the lungs for carbon monoxide using the single-breath method (DLco SB). Lung volume measurements included total lung capacity (TLC) and the ratio of residual volume to total lung capacity (RV/TLC) [29, 35].

### Pulmonary function definitions

We categorized pulmonary function as either normal or impaired. Normal pulmonary function was defined by all measured values falling within reference ranges. Impaired function was diagnosed when any of the following abnormalities were present: obstructive ventilatory dysfunction, restrictive ventilatory disorder, mixed ventilatory disorder, small airway dysfunction, or impaired diffusion capacity. Obstructive ventilatory dysfunction, typically resulting from airway obstruction, was identified by reductions in FEV1 and FEV1/FVC, along with increased RV and TLC [36, 37]. We classified a case as obstructive if the FEV1/FVC ratio was less than 92% of the predicted value, which was automatically calculated based on the subject’s height, weight, age, sex, and race [36, 37]. Restrictive ventilatory disorder, caused by limitations in thoracic or pulmonary expansion, was defined by reductions in FVC and TLC to less than 80% of the predicted value. Mixed ventilatory disorder was diagnosed when features of both obstruction and restriction were present, typically marked by concurrent reductions in FVC, FEV1/FVC, and FEV1. We defined small airway dysfunction as MEF50, MEF25, and MMEF75/25 values below 65% of predicted. When at least two of these three indicators fell below this threshold, the diagnosis of small airway dysfunction was confirmed. Finally, we defined impaired diffusion capacity as a DLco SB value under 80% of the predicted norm [36, 37].

### Diaphragmatic ultrasound in SCA3 patients and healthy controls

To account for potential confounding effects of diaphragmatic dysfunction on pulmonary outcomes, we performed diaphragm ultrasound using a LOGIQ e color Doppler system (GE Healthcare). We measured three key indices: diaphragmatic excursion (DE), diaphragmatic contractile velocity (DCV), and diaphragm thickness (DT). We assessed DE and DCV during quiet breathing, deep breathing, and sniff maneuvers. These parameters reflect diaphragmatic mobility and were used to determine whether diaphragmatic motion remained within physiological limits [38]. DT measurements included inspiratory end thickness during quiet breathing (TEI), expiratory end thickness during quiet breathing (TEE), and TEI during deep inspiration. We also calculated two derived metrics: the diaphragm thickening fraction (DTF) and the diaphragm thickening ratio. These indices allowed us to evaluate both structural integrity and functional contractility of the diaphragm muscle [39]. TEI and TEE values were used to detect signs of diaphragmatic atrophy [39]. The DTF and thickening ratio provided insights into active muscle fiber contraction. We calculated the diaphragm thickening ratio using the following formula: (TEI − TEE) / TEE [39].

### Detection of peripheral inflammatory markers in SCA3 patients and healthy controls

In human participants, “inflammation” refers to systemic inflammatory status indexed by these blood-derived measures. We collected blood samples from all participants under fasting conditions. Routine haematological analyses were conducted using a BC-6800PLUS automated blood analyser (Mindray). The panel included platelet (PLT) count and total WBC count, along with differential counts for neutrophils (NEU), lymphocytes (LYM), monocytes (MON), eosinophils (EOS), and basophils (BAS). We determined WBC, PLT, and red blood cell (RBC) counts using the sheath flow impedance method. Differential classification of white blood cells was performed using semiconductor laser flow cytometry combined with nucleic acid fluorescence staining [40–42]. Cell classification of NEU, LYM, MON, EOS, and BAS was based on a combination of cell volume, internal structural complexity, and nucleic acid fluorescence intensity [22]. To evaluate systemic inflammatory responses, we calculated several leukocyte-derived ratios: NLR, monocyte-to-lymphocyte ratio (MLR), platelet-to-lymphocyte ratio (PLR), lymphocyte-to-monocyte ratio (LMR), and neutrophil-to-platelet ratio (NPR) [20, 21, 23, 43]. In addition, we quantified serum CRP levels using spectrophotometric analysis on the Cobas 8000 C702 automated biochemical analyzer (Roche).

### Breeding and grouping of SCA3 and wild-type mice

We conducted our experiments using hemizygous SCA3-YAC-84Q transgenic mice (SCA3; Strain#: 012705) and wild-type C57BL/6J - (WT; Strain#: 000664) mice, both obtained from the Jackson Laboratory. The SCA3 mouse model, which expresses the full-length human mutant *ATXN3* gene, is widely used in preclinical studies of SCA3 [44–46]. For breeding, we paired male SCA3 mice - aged 8 weeks or older and carrying more than 56 CAG repeats - with age-matched WT females in a 1:1 or 1:2 ratio. The WT females typically delivered pups 19–21 days after mating. We collected tail biopsies from 10-day-old offspring to genotype the *ATXN3* gene via PCR. At approximately 4 weeks of age, we grouped the mice by sex into cages of 3–5 animals, providing unrestricted access to food and water. We maintained the animal facility at 21–23 °C with 40%–60% relative humidity and a 12-hour light/dark cycle. The SCA3 cohort consisted of four mice (1 male and 3 females), while the WT group included three mice (1 male and 2 females). Both groups had an average age of 13.7 months. The mean body weight was 24.7 g for SCA3 mice and 26.2 g for WT mice. Following pulmonary function testing, we euthanized all animals and collected lung tissues for pathological analysis.

### Assessment of pulmonary functions in SCA3 and wild-type mice

We anesthetized all mice and performed tracheal intubation prior to pulmonary testing. Each animal was then placed in the DSI Buxco PFT system (Data Sciences International). Using this system, we conducted three sequential assessments: (1) a functional residual capacity test based on Boyle’s law (FRC test), (2) a quasi-static pressure–volume loop analysis (PV test), and (3) a flow–volume test (FV test). The FRC test measured functional residual capacity (FRC) in each animal. The PV test provided measurements of inspiratory capacity (IC), vital capacity (VC), residual volume (RV) and TLC. The FV test was used to evaluate FVC, forced expiratory volume in 20 milliseconds (FEV20), and the FEV20/FVC ratio.

The Buxco system automatically analyzed and recorded data from each anesthetized mouse within approximately 5 min, allowing for real-time visualization. We compared these measurements across experimental replicates using laminated image overlays to ensure consistency. The FRC test required animals to breathe spontaneously. In contrast, we conducted the PV and FV tests either under spontaneous breathing or with ventilator-assisted respiration.

### Pathological analyses of SCA3 and wild-type mice

Airway-specific inflammation was not directly assessed in humans and was instead evaluated in the SCA3 mice model using lung histopathology and immunohistochemistry. We fixed lungs from each animal in neutral-buffered formalin and embedded them in paraffin. We prepared tissue sections for hematoxylin and eosin (H&E), Masson’s trichrome, or immunohistochemical staining.

For H&E staining, we followed standard histopathological protocols. We first deparaffinized the sections in xylene, then rehydrated them through a graded ethanol series (70%, 80%, 95%, and 100%, each for 1 min). We stained the sections with hematoxylin for 2 min, rinsed them in distilled water, treated them with 0.1% hydrochloric acid in 50% ethanol, and washed them under running tap water for 15 min. We then stained the sections with eosin for 1 min, followed by a final rinse in distilled water. After staining, we dehydrated the slides in 95% and 100% ethanol, cleared them in xylene, and mounted them with coverslips. To investigate whether pulmonary fibrosis contributes to diffuse ventilation impairment in SCA3 mice, we performed Masson’s trichrome staining on lung sections from the SCA3 group. We stained the sections sequentially using Masson’s solution A overnight, then applied a 1:1 mixture of solutions B and C for 1 min. We followed this with solution D for 6 min, solution E for 1 min, and solution F for 2–30 s. After each staining step, we rinsed the slides with 1% glacial acetic acid. We then dehydrated the sections using anhydrous ethanol, cleared them in ethanol and xylene (5 min each), and sealed them with neutral mounting medium.

### Immunohistochemical studies of SCA3 and wild-type mice

We processed paraffin-embedded lung sections from both SCA3 and WT mice by deparaffinizing them in xylene and rehydrating through a graded ethanol series. We then performed antigen retrieval by heating the sections in Tris-EDTA buffer for 30 min. We incubated the sections overnight at 4 °C with the following primary antibodies (all from Servicebio): anti-CD3 (1:500), anti-CD4 (1:500), anti-CD8 (1:500), anti-CD20 (1:1000), anti-CD68 (1:1000), and anti-CD31 (1:1000). To detect the primary antibodies, we applied horseradish peroxidase-conjugated secondary antibodies (Dako). As a negative control, we substituted the primary antibody with a species-matched isotype control. We visualized immunoreactivity using diaminobenzidine (DAB) staining and performed a light hematoxylin counterstain. We quantified the grayscale intensity of the stained sections using ImageJ software (version 1.51k, National Institutes of Health, USA).

### Statistical analysis

We expressed normally distributed continuous variables as mean ± standard deviation, and non-normally distributed variables as median with interquartile range. For comparisons between two groups, we applied an independent-samples *t*-test if both normality and homoscedasticity assumptions were met. When data deviated from a normal distribution, we used the Mann–Whitney U test. We analyzed categorical variables using the Chi-square test.

To evaluate the relationship between genetic and clinical factors and pulmonary function impairment in SCA3 patients, we performed an analysis using binary logistic regression. Pulmonary function status, defined as a binary variable (no impairment versus impairment) based on pulmonary function test results, served as the dependent variable. Age, disease duration, expanded CAG repeat length, and SARA score are independent variables and were included to assess their individual contributions to pulmonary dysfunction.

To examine the link between pulmonary impairment and peripheral inflammation markers in SCA3 patients, we calculated Spearman’s correlation coefficients, which we presented as a heatmap. To control for confounding variables, we constructed multiple linear regression models incorporating sex, smoking history, age, BMI, and inflammation-related markers as independent variables, with pulmonary function parameters as dependent variables. For model stability, we applied a base-10 logarithmic transformation to C-reactive protein (CRP) and basophil counts. In the mouse studies, we compared pulmonary function parameters and immunohistochemical outcomes using either independent-samples *t-*test or Mann–Whitney U test, depending on data normality and variance homogeneity. We set statistical significance at *P* < 0.05. All analyses were conducted using SPSS version 24.0, and graphical representations were generated with GraphPad Prism 8.0.

## Results

### Demographic characteristics of participants

We recruited 102 patients with genetically confirmed SCA3 and 91 age-matched healthy controls. No significant differences were observed between the two groups in terms of smoking history, age, sex distribution, height, or BMI. In the SCA3 group, the mean AAO was 35.89 ± 11.10 years. The median number of expanded CAG repeats was 75.00 (interquartile range: 72.00–77.00), with a median disease duration of 6.00 years (4.00–10.00) and a median SARA score of 9.50 (7.50–12.00) (Table 1).

**Table 1:** Baseline characteristics of patients with SCA3 and healthy controls

	HCs	SCA3	*P*-Value
*N*	91	102	
Smoking history, yes/no	27/64	43/59	0.072^1^
Gender, M/F	50/41	63/39	0.337^1^
Age, years	44.89 ± 13.09	45 (35–51)	0.463^2^
Height, centimeters	165 (160–171)	164.17 ± 8.07	0.195^2^
BMI, kg/m^2^	22.24 ± 2.19	21.95 ± 3.29	0.470^3^
AAO, years	NA	35.89 ± 11.10	
Duration, years	NA	6.00 (4.00–10.00)	
Expanded CAG repeats	NA	75.00 (72.00–77.00)	
SARA	NA	9.50 (7.50–12.00)	

Variables with normal distribution are presented as means ± SD; variables in non-normal distributions are expressed as median (range)

HCs = healthy controls; AAO = age at onset; BMI = body mass index; NA = not applicable; SARA = scale for the assessment and rating of ataxia

^1^Chi-square test

^2^Mann-Whitney U test

^3^Independent-samples *t*-test

### Impaired pulmonary function in SCA3 patients

Pulmonary function testing revealed that SCA3 patients exhibited significant reductions in several parameters compared to controls, including FVC, FEV1, PEF, MEF75, MEF50, MEF25, MMEF75/25, MVV, TLC%, VC MAX, and DLco SB. Conversely, the RV/TLC ratio was significantly elevated. No significant differences were detected between groups for FEV1/FVC or FEF50/FIF50 ratios (Table 2). These findings suggest mild restrictive ventilatory defects, small airway dysfunction, and impaired gas diffusion capacity, without evidence of upper airway obstruction or overt obstructive ventilatory impairment. Given the absence of respiratory symptoms, these abnormalities indicate subclinical pulmonary dysfunction.

**Table 2:** Comparison of pulmonary function in SCA3 patients and healthy controls

	HCs	SCA3	*P*-Value
FVC, % predicted	103.79 ± 11.17	95.85 (85.55–102.74)	< 0.001^1^
FEV1, % predicted	101.90 (96.20–109.80)	93.03 ± 14.67	< 0.001^1^
FEV1/FVC	83.35 ± 4.27	83.60 ± 7.32	0.764^2^
PEF, % predicted	106.74 ± 14.53	91.20 (79.93–101.52)	< 0.001^1^
MEF75, % predicted	109.52 ± 17.04	94.97 ± 22.00	< 0.001^2^
MEF50, % predicted	92.20 (83.20–106.80)	86.89 ± 24.55	0.004^1^
MEF25, % predicted	73.00 (61.90–94.40)	67.55 (53.17–90.23)	0.012^1^
MMEF75/25, % predicted	86.20 (76.10–99.90)	78.99 ± 24.29	< 0.001^1^
FEF50/FIF50	112.58 (93.28–153.32)	130.10 (101.30–179.99)	0.086^1^
FET, s	6.12 (4.29–6.71)	5.97 (3.64–6.61)	0.199^1^
MVV, % predicted	98.40 (89.00–105.38)	72.77 ± 20.60	< 0.001^1^
TLC, % predicted	97.13 (91.50–103.30)	94.28 ± 11.98	0.031^1^
RV/TLC	35.26 ± 6.83	38.57 (34.29–43.39)	< 0.001^1^
DLco SB, % predicted	88.08 ± 13.72	82.14 (74.85–94.77)	0.015^1^
VC MAX, % predicted	101.23 ± 10.89	93.54 (84.73–100.48)	< 0.001^1^
Non-impaired vs. Impaired	91/0	31/71	< 0.001^3^

Variables with normal distribution are presented as means ± SD; variables in non-normal distributions are expressed as median (range)

HCs = healthy controls; FVC = forced vital capacity; FEV1 = forced expiratory volume in 1 s; FEV1/FVC = forced expiratory volume in 1 s to forced vital capacity ratio; PEF = peak expiratory flow; MEF75 = maximal expiratory flow at 75% vital capacity; MEF50 = maximal expiratory flow at 50% vital capacity; MEF25 = maximal expiratory flow at 25% vital capacity; MMEF75/25 = maximal mid-expiratory flow (25–75%); FEF50/FIF50 = forced expiratory flow at 50% vital capacity to forced inspiratory flow at 50% vital capacity ratio; FET = forced expiratory time; MVV = maximal voluntary ventilation; TLC = total lung capacity; RV/TLC = residual volume to total lung capacity ratio; DLco SB = diffusion capacity of the lung for carbon monoxide, single-breath; VC MAX = maximal vital capacity

^1^Mann-Whitney U test

^2^Independent-samples *t*-test

^3^Chi-square test

Of the 102 SCA3 patients, 71 were categorized as having impaired pulmonary function, while the remaining 31 were classified as non-impaired. All 91 healthy controls fell into the non-impaired category. This distribution was statistically significant (*P* < 0. 001) (Table 2).

### Impaired pulmonary function is correlated with ataxia severity

We performed a binary logistic regression analysis to assess whether clinical and genetic factors predicted pulmonary function status (impaired vs. unimpaired) in SCA3 patients. Independent variables included SARA score, age, disease duration, and expanded CAG repeat number. The analysis identified the SARA score as the only significant predictor. Higher SARA scores were associated with an increased risk of pulmonary impairment (OR = 1.196, 95% CI: 1.038–1.376, *P* = 0.013) (Supplementary Table 1).

### Normal diaphragm function in SCA3 patients

To determine whether diaphragm dysfunction contributes to pulmonary impairment in SCA3, we examined diaphragm function in a subset of 27 SCA3 patients and 8 healthy controls. No significant differences were observed between groups in DE, DCV, or DT (*P* > 0.05). As DE and DCV reflect diaphragmatic mobility and function, and DT serves as a marker of diaphragmatic atrophy, these findings indicate preserved diaphragm function in SCA3 patients. Thus, the pulmonary dysfunction observed in this cohort does not appear to result from diaphragmatic involvement (Supplementary Table 2).

### Impaired pulmonary function associated with peripheral inflammatory markers

We analyzed routine blood parameters in 88 SCA3 patients and 88 age- and sex-matched healthy controls. For CRP measurements, we included 77 matched pairs. Demographic data are presented in Table 3.

**Table 3:** Demographic characteristics of enrolled individuals

	HCs	SCA3	*P*-Value
Individuals with blood cell count–derived inflammatory indices			
N	88	88	
Age, years	43.00 ± 11.17	44.50 (34.00, 51.00)	0.905^1^
Gender, M/F	55/33	55/33	1.000^2^
Leukocytes, ×10^9^/L	5.20 ± 0.94	6.63 (5.65, 7.52)	< 0.001^1^
Neutrophils, ×10^9^/L	2.85 ± 0.71	3.87 (3.13, 4.59)	< 0.001^1^
Lymphocytes, ×10^9^/L	1.69 (1.48, 2.04)	2.07 ± 0.55	< 0.001^1^
Monocytes, ×10^9^/L	0.25 (0.21, 0.29)	0.33 (0.26, 0.42)	< 0.001^1^
Eosinophils, ×10^9^/L	0.13 (0.08, 0.17)	0.13 (0.07, 0.20)	0.768^1^
Basophils, ×10^9^/L	0.03 (0.02, 0.04)	0.03 (0.02, 0.05)	0.040^1^
PLT, ×10^9^/L	221.36 ± 53.54	255.00 (220.25, 281.75)	< 0.001^1^
NLR, ratio	1.68 ± 0.54	2.05 ± 0.88	0.001^3^
MLR, ratio	0.15 ± 0.04	0.16 (0.13, 0.20)	0.011^1^
PLR, ratio	121.76 (102.90, 151.69)	121.81 (105.83, 157.66)	0.597^1^
LMR, ratio	6.88 (5.57, 8.41)	6.43 ± 2.14	0.011^1^
NPR, ratio	0.01 (0.01, 0.02)	0.01 (0.01, 0.02)	0.009^1^
Individuals with CRP index			
N	77	77	
Age, years	43.55 ± 10.18	43.22 ± 11.22	0.851^3^
Gender, M/F	48/29	48/29	1.000^2^
CRP, mg/L	1.10 (0.52, 1.90)	0.59 (0.30, 1.15)	0.001^1^

Variables with normal distribution are presented as means ± SD; variables in non-normal distributions are expressed as median (range)

HCs = healthy controls; NLR = neutrophil to lymphocyte ratio; MLR = monocyte to lymphocyte ratio; PLR = platelet to lymphocyte ratio; LMR = lymphocyte to monocyte ratio; NPR = neutrophil to platelet ratio

^1^Mann-Whitney U test

^2^Chi-square test

^3^Independent samples *t*-test

Our results revealed that, except for eosinophils and the PLR (*P* > 0.05), SCA3 patients exhibited significantly elevated levels of peripheral inflammation-related markers compared to controls. In the SCA3 group, several inflammatory markers correlated with pulmonary function indices. Neutrophil counts were inversely associated with MEF25 (ρ = − 0.230, *P* = 0.031). Similarly, the NLR showed negative correlations with MEF25 (ρ = − 0.272, *P* = 0.010) and MMEF75/25 (ρ = − 0.243, *P* = 0.023). NPR also demonstrated inverse associations with MEF25 (ρ = − 0.255, *P* = 0.016) and MMEF75/25 (ρ = − 0.227, *P* = 0.034). In contrast, basophil counts correlated positively with PEF (ρ = 0.215, *P* = 0.045) (Supplementary Fig. 1). Multivariate linear regression analysis showed that NPR remained an independent negative predictor of MEF25 (β = − 1025.094, *R²* = 0.121) after adjusting for potential confounders. However, neutrophil count, basophil count, and NLR lost statistical significance in these adjusted models. Importantly, CRP emerged as a strong negative predictor of several key pulmonary parameters, including FVC (β = − 3.662, *R²* = 0.261), FEV1 (β = − 3.875, *R²* = 0.228), and VC MAX (β = − 3.451, *R²* = 0.294). Conversely, eosinophil levels showed significant positive associations with FVC (β = 33.917, *R²* = 0.254) and VC MAX (β = 32.029, *R²* = 0.287) (Supplementary Table 3).

### Impaired pulmonary function of SCA3 mice

**Fig. 1:**
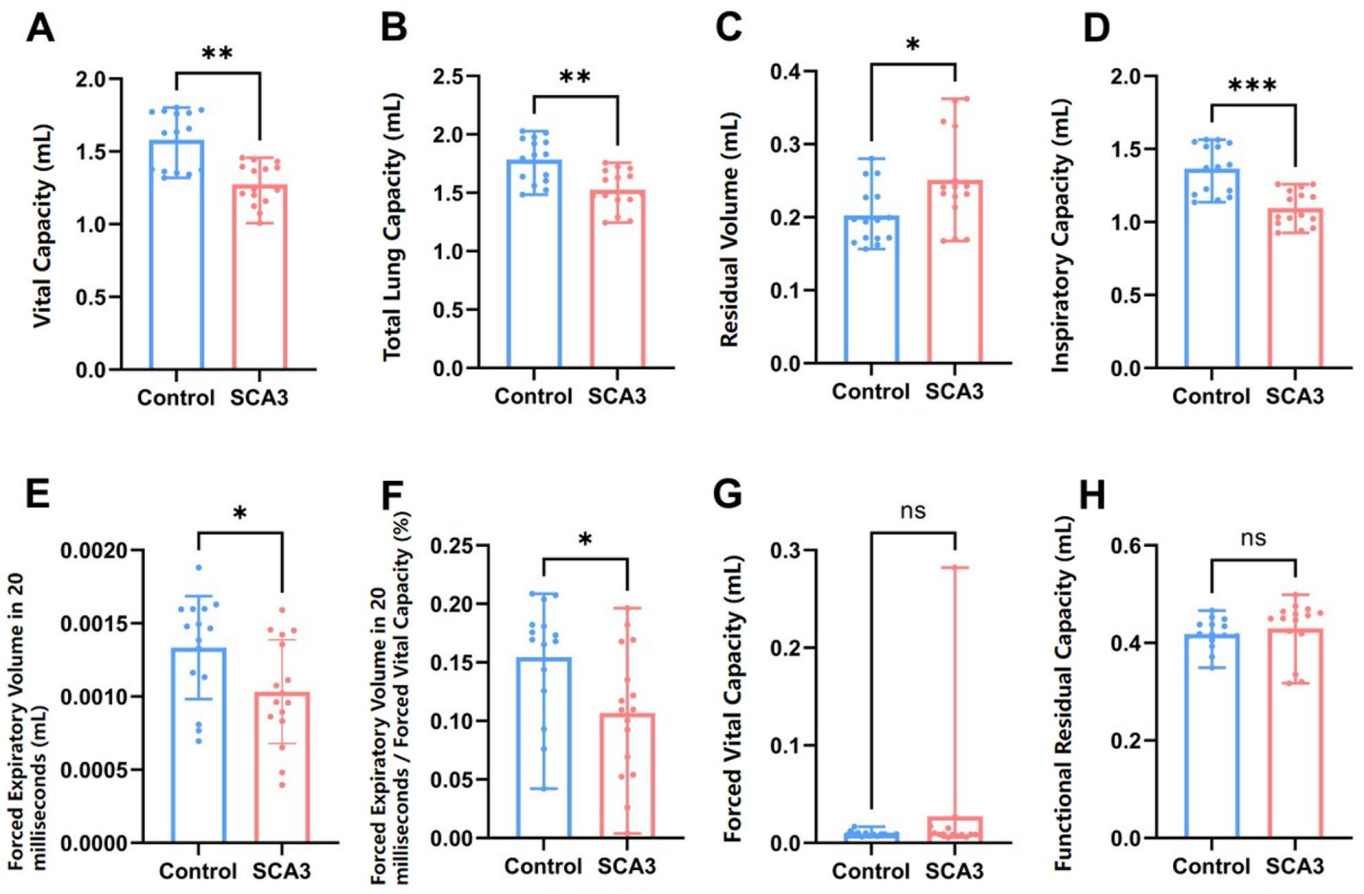
Mixed ventilation dysfunction in SCA3 model mice. **A**-**F**: Compared to the wild-type control group, the SCA3 group showed significant decreases for VC, TLC, IC, FEV20, and FEV20/FVC, while RV increased. **G**, **H**: No differences in FVC or FRC were observed between SCA3 and WT control groups. All indicators of each mouse were tested 3 to 6 times. ^*^P < 0.05, ^**^P < 0.01, ^***^P < 0.001. ns, non-significant

We conducted pulmonary function tests on seven thirteen-month-old mice, including four SCA3 transgenic mice and three WT controls. Compared to WT mice, SCA3 mice exhibited significant reductions in VC, TLC, IC, FEV20, and the FEV20/FVC ratio. Additionally, RV was significantly increased. No differences were observed in FVC or FRC between groups (Fig. 1). The decreased FEV20 and FEV20/FVC ratio, along with elevated RV, indicate obstructive ventilatory impairment in the SCA3 mice. Simultaneously, the reductions in VC, TLC, and IC reflect restrictive ventilatory dysfunction. These findings demonstrate that the transgenic SCA3 model exhibits mixed ventilatory impairment.

### Chronic inflammation in small airways of SCA3 mice

**Fig. 2:**
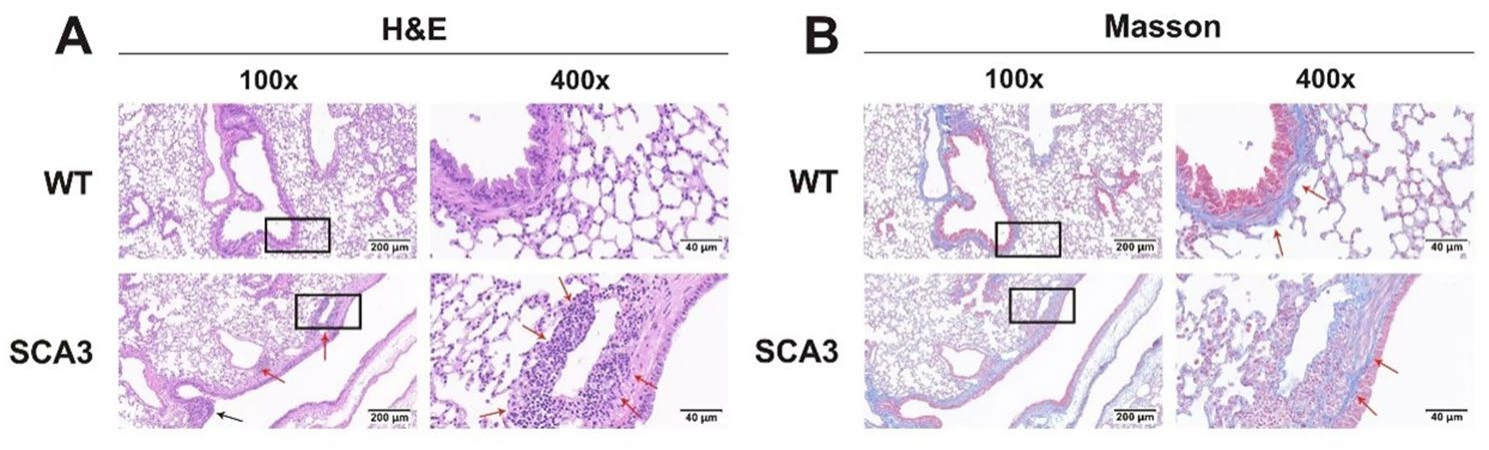
Chronic inflammation around small airways and blood vessels in SCA3 mice. **A**: Compared to the wild-type control group, HE staining showed significant infiltration of inflammatory cells around the small airways (black arrows) and blood vessels (red arrows) in the SCA3 group (X100 and X400 magnifications). **B**: Masson staining indicated no obvious differences in the degree of fibrosis (red arrows) between the SCA3 and WT control groups (X100 and X400 magnifications)

Hematoxylin and eosin (H&E) staining demonstrated that WT mice had preserved lung architecture, with intact bronchial structures, uniformly distributed vasculature, and no evident pathological abnormalities (Fig. 2). In contrast, lung sections from SCA3 mice displayed thickening of small airway and vascular walls, primarily due to inflammatory cell infiltration. Masson’s trichrome staining showed no significant difference in pulmonary fibrosis between the SCA3 and WT groups. These findings suggest that the diffuse ventilatory dysfunction observed in SCA3 mice is associated with airway inflammation rather than fibrotic remodeling.

**Fig. 3:**
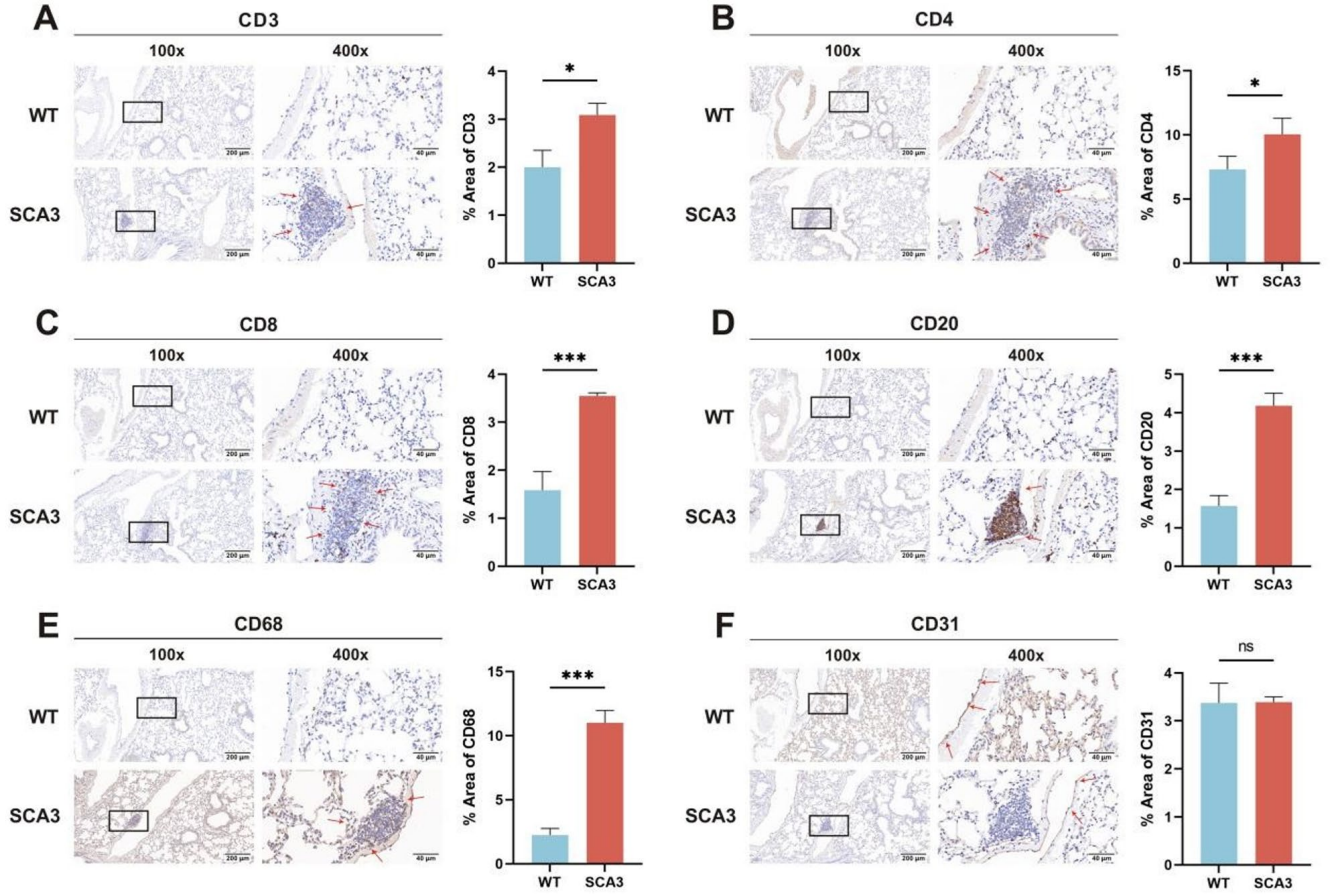
Lymphocytes and macrophages are involved in small airway inflammatory changes in SCA3 mice. **A**-**C**: Immunohistochemistry confirms that CD3^+^, CD4^+^, and CD8^+^ (red arrows) are not expressed in lung sections of the WT group, while the expression around small airways and blood vessels in the SCA3 group was significantly increased (X100 and X400 magnifications). **D**, **E**: Only a small amount of CD20^+^ and CD68^+^ (red arrows) expression was observed in the WT group, while the expression of CD20^+^ and CD68^+^ in the small airways and perivascular areas significantly increased in the SCA3 group (X100 and X400 magnifications). **F**: CD31^+^ (red arrows) suggests vascular localization (X100 and X400 magnifications). ^*^P < 0.05, ^***^P < 0.001. ns, non-significant

To further characterize peribronchiolar and perivascular inflammation, we performed immunohistochemical (IHC) analysis on lung sections from both SCA3 and WT mice. We used antibodies targeting T lymphocytes (CD3 for pan-T cells, CD4 for helper T cells, CD8 for cytotoxic T cells), B lymphocytes (CD20), macrophages (CD68), and vascular endothelial cells (CD31). IHC revealed significantly greater peribronchiolar and perivascular immune cell infiltration in SCA3 mice compared to WT controls. Specifically, the immune-positive areas for CD3⁺ (*P* < 0.05), CD4⁺ (*P* < 0.05), CD8⁺ (*P* < 0.001), CD20⁺ (*P* < 0.001), and CD68⁺ (*P* < 0.001) were markedly elevated in SCA3 lung tissues (Fig. 3).

## Discussion

This study compared pulmonary function parameters between 102 SCA3 patients and 91 healthy controls, revealing subclinical pulmonary impairment in SCA3 characterized by restrictive ventilation deficits, small airway dysfunction, and reduced diffusion capacity. Ataxia severity positively correlated with pulmonary dysfunction. Although diaphragmatic movement remained unaffected, pulmonary impairment in SCA3 patients was associated with elevated peripheral inflammatory markers. In the SCA3 mice model, pulmonary impairment also correlated with peribronchiolar/perivascular inflammatory infiltration. Taken together, this evidence suggests an inflammatory component to small airway involvement.

These results indicate that pulmonary decline occurs early in neurologically symptomatic SCA3 patients, even before respiratory symptoms arise. Similarly, studies on Friedreich’s ataxia (FRDA) report restrictive respiratory impairment, with reductions in FVC, airflow, and respiratory pressures; more than half of FRDA patients also exhibit decreased maximal expiratory pressure (MEP) and maximal inspiratory pressure (MIP) [47]. Smaller studies have documented reduced pulmonary parameters - including MVV, MEP, and MIP - in SCA3 patients, while other SCA subtypes show declines in FVC, peak expiratory flow rate (PEFR), MVV, MIP, and MEP, alongside elevated FEV1/PEFR ratios, consistent with subclinical restrictive ventilatory dysfunction [19]. Our findings corroborate these reports across multiple SCA subtypes. However, the mechanisms driving pulmonary impairment in SCA remain unclear. Although diaphragmatic ultrasound effectively assesses respiratory function in ALS by measuring diaphragm thickness and thickening ratio [48], our data show preserved diaphragmatic function in SCA3 patients. This suggests that pulmonary dysfunction in SCA3 arises from mechanisms other than diaphragmatic impairment.

Our analysis revealed that impaired pulmonary function in SCA3 patients correlates with elevated levels of CRP and the NPR. CRP, a nonspecific acute-phase protein, showed a significant inverse relationship with FVC, FEV1, and VC MAX after controlling for potential SCA3 risk factors, although its source - whether infectious or inflammatory - remains uncertain. Concurrently, NPR correlated negatively with MEF25, suggesting its potential as a biomarker for small airway dysfunction. Prior studies have established strong links between pulmonary function and blood cell profiles, including neutrophils, eosinophils, and basophils [22]. However, to our knowledge, no large-scale population studies have examined whether NPR independently predicts impaired lung function in SCA3 or other pulmonary diseases. NPR may offer a simple, accessible metric reflecting the impact of external exposures on respiratory health, but further investigation is necessary to validate its clinical utility in this context.

Growing evidence suggests that neuroimmune modulation may play a central role in pulmonary inflammation. Neurotransmitters and neuropeptides can directly interact with alveolar macrophages, stimulating cytokine production and initiating neurogenic inflammation [49]. In SCA3, the accumulation of toxic Poly Q aggregates from mutant ataxin-3 causes neuronal degeneration [50]; however, its immunomodulatory effects remain largely unexplored. Recent studies have identified mast cells as key mediators of neuroinflammatory responses [51]. Specifically, in the brain tissues of SCA3 mice, expanded ataxin-3 has been shown to downregulate pro-inflammatory cytokine TNF-α, the NF-κB inhibitor IκBα, and the mast cell marker CD117/c-Kit, while upregulating the chemokine CXCL1 [52], which recruits granulocytes. Additionally, *ATXN3* has been implicated in amplifying type I interferon (IFN-I)-mediated inflammation in both murine lung tissue and human epithelial cells [53]. Based on these findings, we propose that chronic small airway inflammation in SCA3 may involve Poly Q-induced neuroimmune interactions within pulmonary tissue; this hypothesis warrants further experimental validation.

This study has limitations. Firstly, it is a cross-sectional analysis of pulmonary function, lacking longitudinal data to monitor changes over time. Next, our cohort did not include individuals with pre-ataxic SCA3, which limits insight into early disease progression. The animal experiments also only included a relatively small sample, which may limit statistical power and reproducibility. In addition, as swallowing function and aspiration risk were not systematically assessed, we cannot exclude the possibility that subclinical dysphagia may be relevant in some patients. Pulmonary infections likewise significantly affect pulmonary function test results, as seen in cases of chronic Pseudomonas aeruginosa infection [54]. Future experiments will include an assessment of swallowing function and lung CT. Finally, medication use and physical activity levels were not systematically captured and may also influence pulmonary function and systemic inflammatory markers. The Poly Q-related neuroimmune pathway therefore remains a mechanistic hypothesis that warrants further longitudinal and experimental validation.

## Conclusion

Pulmonary dysfunction in SCA3 patients is associated with disease severity. Our findings suggest that chronic inflammation in the small airways may be involved in the decline in respiratory function. Routine pulmonary function testing could serve as a valuable screening tool for the early identification and management of subclinical lung inflammation, potentially improving long-term outcomes in individuals with SCA3.

## Electronic Supplementary Material

Below is the link to the electronic supplementary material.

Supplementary Material 1

## Data Availability

All data generated or analyzed during this study are included in this published article and its supplementary information files.
