# Supplementary material for: Relationship of subclinical lung injury to chronic airway inflammation in spinocerebellar ataxia type 3
Source: Orphanet J Rare Dis. 2026 Mar 9;21:154. doi: 10.1186/s13023-026-04306-5 (PMC13085427; doi:10.1186/s13023-026-04306-5)
Supplement: Supplementary file 1 — Supplementary Material 1 [file 13023_2026_4306_MOESM1_ESM.docx]

**Supplementary figure**

**Supplementary Figure 1**

**Heatmaps showing Spearman correlation coefficients of pulmonary function parameters and inflammatory-related indices.**

**
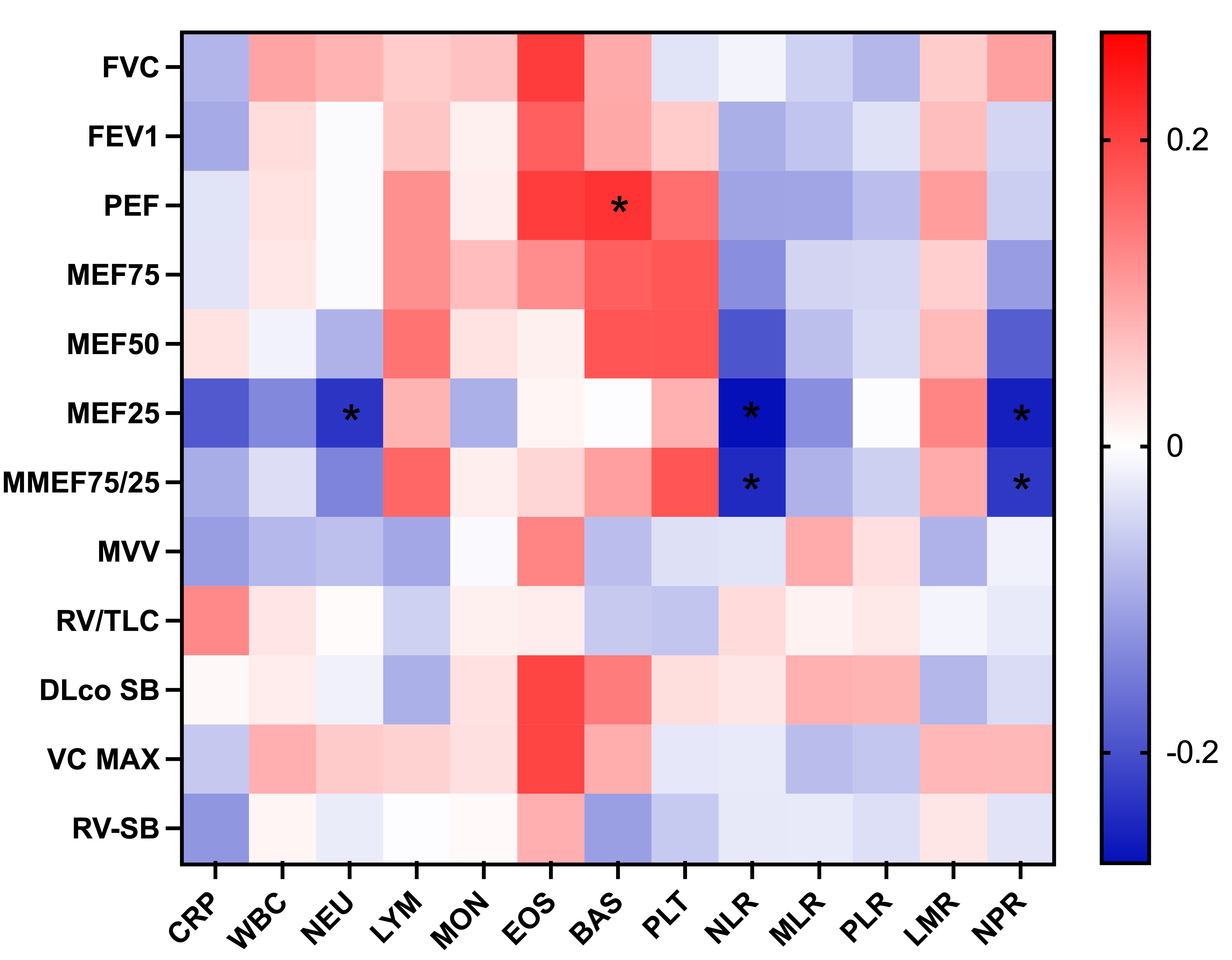
**

Spearman's correlation analysis showing a negative correlation between neutrophils and MEF25 (ρ = -0.230; *P* = 0.031), a positive correlation between basophils and PEF (ρ = 0.215, *P* = 0.045), and a negative correlation between NLR and both MEF25 and MMEF75/25 (ρ = -0.272, *P* = 0.010; ρ= -0.243, *P* = 0.023). NPR was negatively correlated with MEF25 and MMEF75/25 (ρ = -0.255, *P* = 0.016; ρ = -0.227, *P* = 0.034). ^*^*P* < 0.05.

**Supplementary tables**

**Supplementary Table 1**

**Correlation between clinical manifestations and pulmonary function in SCA3 patients**

|  | β | S.E. | Wald χ^2^ | Sig. | OR | 95% CI |
| --- | --- | --- | --- | --- | --- | --- |
| Age, years | 0.029 | 0.029 | 0.956 | 0.328 | 1.029 | 0.972, 1.090 |
| Disease duration, years | -0.031 | 0.046 | 0.457 | 0.499 | 0.969 | 0.886, 1.061 |
| Expanded CAG repeats | 0.075 | 0.075 | 0.988 | 0.32 | 1.078 | 0.930, 1.248 |
| SARA | 0.179 | 0.072 | 6.174 | 0.013^*^ | 1.196 | 1.038, 1.376 |
| Constant | -7.475 | 6.403 | 1.363 | 0.243 | 0.001 |  |

SARA = scale for the assessment and rating of ataxia. ^*^P < 0.05.

**Supplementary Table 2**

**Diaphragm function in SCA3 patients and healthy controls**

|  |  | HCs | SCA3 | *P*-Value |
| --- | --- | --- | --- | --- |
|  | Number | 8 | 27 |  |
|  | Gender, M/F | 2/6 | 15/12 | 0.228^1^ |
|  | Age, years | 41.0 ± 10.66 | 38.78 ± 10.53 | 0.604^2^ |
| DE, cm | Quiet breath | 1.63 ± 0.63 | 1.23 (0.87–1.44) | 0.067^3^ |
|  | Deep breath | 4.02 ± 0.91 | 3.46 (2.97–4.21) | 0.343^3^ |
|  | Sniff test | 3.02 ± 1.05 | 2.41 ± 0.90 | 0.117^2^ |
| DCV, cm/s | Quiet breath | 1.46 ± 0.58 | 0.99 (0.83–1.27) | 0.143^3^ |
|  | Deep breath | 2.17 ± 0.71 | 2.06 ± 0.83 | 0.754^2^ |
|  | Sniff test | 5.61 ± 2.16 | 4.66 ± 2.16 | 0.283^2^ |
| DT, cm | TEI during quiet breathing | 0.18 ± 0.03 | 0.16 (0.13–0.22) | 0.451^3^ |
|  | TEE during quiet breathing | 0.15 ± 0.03 | 0.13 (0.11–0.15) | 0.192^3^ |
|  | TEI during deep breathing | 0.29 ± 0.06 | 0.29 ± 0.11 | 0.844^2^ |
|  | DTF | 0.22 ± 0.07 | 0.30 (0.18–0.45) | 0.104^3^ |
|  | Diaphragm thickening ratio | 1.22 ± 0.07 | 1.30 (1.18–1.45) | 0.104^3^ |

Variables with normal distribution are presented as means ± SD; variables in non-normal distributions are expressed as median (range).

HCs = healthy controls; DE = diaphragmatic excursion; DCV = diaphragm contraction velocity; DT = diaphragm thickness; TEI = end-inspiratory thickness; TEE = end-expiratory thickness; DTF = diaphragmatic thickening fraction.

^1^[Chi-square test](https://www.bing.com/ck/a?!&&p=021c9abbc9b2b9caJmltdHM9MTcyMTA4ODAwMCZpZ3VpZD0wYjMzNTdkOS1mOGQ3LTZjYzMtMDAxOC00M2MyZjliMTZkZmEmaW5zaWQ9NTQ1Nw&ptn=3&ver=2&hsh=3&fclid=0b3357d9-f8d7-6cc3-0018-43c2f9b16dfa&u=a1aHR0cHM6Ly9ibG9nLmNzZG4ubmV0L3pmY2poZHEvYXJ0aWNsZS9kZXRhaWxzLzgzNTEyNjgw&ntb=1)

^2^Independent-samples *t*-test

^3^Mann-Whitney U test

| **Supplementary Table 3**  **Multiple linear regression analyses between pulmonary function parameters and peripheral inflammation-related markers in SCA3 patients** | | | | | | |
| --- | --- | --- | --- | --- | --- | --- |
| Dependent variables | Independent variable | β | Std. error | *T*-Value | *P*-Value | VIF |
| FVC,  % predicted | Gender, M/F | 8.910 | 4.104 | 2.171 | 0.033^*^ | 1.581 |
|  | Smoking history, yes/no | 5.160 | 4.099 | 1.259 | 0.212 | 1.631 |
|  | Age, years | 0.573 | 0.152 | 3.765 | <0.001^***^ | 1.152 |
|  | BMI, kg/m^2^ | 0.878 | 0.556 | 1.578 | 0.119 | 1.123 |
|  | lg ^CRP^ | -3.662 | 1.735 | -2.111 | 0.038^*^ | 1.203 |
| FEV1,  % predicted | Gender, M/F | 6.957 | 3.954 | 1.760 | 0.083 | 1.581 |
|  | Smoking history, yes/no | 5.178 | 3.948 | 1.311 | 0.194 | 1.631 |
|  | Age, years | 0.446 | 0.147 | 3.043 | 0.003^**^ | 1.152 |
|  | BMI, kg/m^2^ | 1.091 | 0.536 | 2.036 | 0.045^*^ | 1.123 |
|  | lg ^CRP^ | -3.875 | 1.671 | -2.319 | 0.023^*^ | 1.203 |
| VC MAX,  % predicted | Gender, M/F | 9.526 | 3.789 | 2.514 | 0.014^*^ | 1.581 |
|  | Smoking history, yes/no | 4.246 | 3.784 | 1.122 | 0.266 | 1.631 |
|  | Age, years | 0.549 | 0.141 | 3.910 | < 0.001^***^ | 1.152 |
|  | BMI, kg/m^2^ | 1.030 | 0.513 | 2.006 | 0.049^*^ | 1.123 |
|  | lg ^CRP^ | -3.451 | 1.602 | -2.155 | 0.035^*^ | 1.203 |
| FVC,  % predicted | Gender, M/F | 11.250 | 3.610 | 3.116 | 0.003^**^ | 1.459 |
|  | Smoking history, yes/no | 4.954 | 3.711 | 1.335 | 0.186 | 1.542 |
|  | Age, years | 0.429 | 0.132 | 3.250 | 0.002^**^ | 1.063 |
|  | BMI, kg/m^2^ | 0.853 | 0.460 | 1.853 | 0.068 | 1.058 |
|  | Eosinophils, ×10^9^/L | 33.917 | 16.687 | 2.033 | 0.045^*^ | 1.136 |
| VC MAX,  % predicted | Gender, M/F | 12.304 | 3.345 | 3.679 | <0.001^***^ | 1.459 |
|  | Smoking history, yes/no | 4.463 | 3.438 | 1.298 | 0.198 | 1.542 |
|  | Age, years | 0.412 | 0.122 | 3.364 | 0.001^**^ | 1.063 |
|  | BMI, kg/m^2^ | 0.921 | 0.427 | 2.159 | 0.034^*^ | 1.058 |
|  | Eosinophils, ×10^9^/L | 32.029 | 15.461 | 2.072 | 0.041^*^ | 1.136 |
| MEF25,  % predicted | Gender, M/F | -4.943 | 7.585 | -0.652 | 0.516 | 1.449 |
|  | Smoking history, yes/no | 2.929 | 7.638 | 0.384 | 0.702 | 1.470 |
|  | Age, years | -0.616 | 0.281 | -2.194 | 0.031^*^ | 1.079 |
|  | BMI, kg/m^2^ | -0.305 | 0.965 | -0.316 | 0.753 | 1.045 |
|  | NPR, ratio | -1025.094 | 489.778 | -2.093 | 0.039^*^ | 1.028 |

VC MAX = vital capacity maximum; BMI = body mass index; CRP = C-reactive protein; NPR = neutrophil to platelet ratio. ^*^P < 0.05, ^**^P < 0.01, ^***^P < 0.001.
